# Identification of hemolysin encoding genes and their association with antimicrobial resistance pattern among clinical isolates of coagulase-negative *Staphylococci*

**DOI:** 10.1186/s13104-020-4938-0

**Published:** 2020-02-10

**Authors:** Mona Nasaj, Zahra Saeidi, Babak Asghari, Ghodratollah Roshanaei, Mohammad Reza Arabestani

**Affiliations:** 1 Department of Microbiology, Faculty of Medicine, Hamadan University of Medical Sciences, Shahid Fahmideh Street, Park Mardome, Hamadan, IR Iran; 2 Department of Biostatistics, School of Health, Hamadan University of Medical Sciences, Shahid Fahmideh Street, Park Mardome, Hamadan, IR Iran; 3 Nutrition Research Center, Hamadan University of Medical Sciences, Shahid Fahmideh Street, Park Mardome, Hamadan, IR Iran

**Keywords:** CoNS, Hemolysin, Antibiotic resistance

## Abstract

**Objective:**

Coagulase-negative *staphylococci* (CoNS) are as considered opportunistic pathogens which capable of producing several toxins, enzymes and resistance genes. The current study aimed to determine the occurrence of different hemolysins genes and patterns of antibiotic resistance among CoNS species.

**Results:**

The highest frequency of antibiotic resistance was observed against cefoxitin in 49 isolates (53.8%), and the lowest resistance was against novobiocin in 5 isolates (5.5%). None of the isolates was resistant to vancomycin. The prevalence of *hla, hla_yidD, hld,* and *hlb* genes was: 87.9%, 62.6%, 56%, and 47.3%, respectively. The *hla/yidD* and *hld* genes were detected in 69.4% of *S. epidermidis* and the *hla* gene in 94.6% of *S. haemolyticus* isolates; the *hlb* gene was detected in 53.1% of the *S. epidermidis* isolates. The *mecA* gene was identified in 50 (55%) of the CoNS isolates. In conclusion, the results of statistical analysis showed that the *hld gene* had a significant association with resistance to levofloxacin and erythromycin antibiotics, the *hlb* with clindamycin resistance and the *hla/yidD* with rifampicin and novobiocin resistance. The results of this study showed that there is a significant relationship between hemolysin encoding genes and antibiotic resistance patterns; therefore, detection of virulence factors associated with antibiotic resistance has become a significant issue of concern.

## Introduction

Coagulase-negative *staphylococci* (CoNS) are considered as opportunistic pathogens that cause a variety of infections, particularly among immunocompromised, long-term hospitalized patients, preterm infants and in patients with indwelling or different implant polymer bodies [1–3]. Among various CoNS species, *Staphylococcus epidermidis, Staphylococcus haemolyticus*, and *Staphylococcus saprophyticus* have been confirmed to be responsible agents for the vast majority of nosocomial infections [4]. Treatment of CoNS infections has become more complicated, as many isolates in hospitals show high rates of resistance to multiple antimicrobial agents of clinical relevance [5]. CoNS is also a reservoir for resistance genes that can be transmitted to other pathogens [6]. About 80–90% of CoNS isolates associated with nosocomial infections are methicillin-resistant coagulase-negative *Staphylococci* [7]. Most antibiotic resistance genes are carried on a plasmid and more often found in methicillin-resistant [8]. CoNS are capable of producing several toxins and enzymes characteristically associated with *Staphylococcus aureus* such as hemolysins, which are responsible for the invasion of host cells [9, 10]. Hemolysins of *staphylococci* are categorized into four different types, including alpha (α), beta (β), gamma (γ), and delta (δ). The α-toxin is encoded by the *hla* and acts as a pore-forming cytotoxin (PFT) which actives against a wide array of human cells. Pathogenicity of this toxin is due to hemolytic, dermonecrotic, and neurotoxic effects [11–14]. β-toxin which encoded by the *hlb* gene is known as Mg^2+^ -dependent sphingomyelinase. Incubation at temperatures below 10 °C intensifies the cytolytic activity of β-toxin; thus, it is often referred to as the ‘hot–cold’ hemolysin [15, 16]. The *hld* gene encodes a 26 amino acid peptide, which is referred to as delta (δ) hemolysin. This toxin with its detergent function degrades erythrocytes. The delta toxin may cause intestinal diseases that can vary from acute diarrhea to severe enteritis [15, 17, 18]. Because the association between antimicrobial resistance and virulence factors of CoNS isolates had never been done before as well as in a similar work in *staphylococcus aureus* strains observed a significant relationship between antibiotic resistance and hemolysin genes thus we made attempt to determine this association in this study.

## Main text

### Methods

#### Identification of CoNS isolates

A total of the 91 CoNS isolates were collected from various clinical specimens submitted to three teaching hospitals (including Beheshti, Besat, and Farshchian) located in Hamedan, Iran, from September 2017 to November 2018. All isolates were investigated by conventional methods. The origins of the isolates were as follows: blood, urine, catheters, and wounds. This study was approved by the Ethics Committee of Hamadan University of Medical Sciences (Code No: IR.UMSHA.REC.1396.827).

#### Antibiotic susceptibility testing

The Antibiotic susceptibility testing of 91 CoNS species carried out using the standard disk agar diffusion (DAD) method according to the Clinical and Laboratory Standards Institute (CLSI) guidelines [19]. Mueller–Hinton agar culture medium (Merck, Germany) was inoculated with a saline suspension of isolated CoNS equivalent to McFarland 0.5 standard. After that, antibiotic discs were placed on the surface of the agar. After 16-18 h incubation at 37 °C, CoNS isolates were categorized to be resistant, intermediately resistant, or sensitive based on the size of growth inhibition zone. The antimicrobial agents used in current study were as follows: for the following antimicrobial agents: chloramphenicol (30 µg), cefoxitin (30 µg), clindamycin (2 µg), doxycycline (30 µg), erythromycin (15 µg), gentamicin (10 µg), levofloxacin (5 µg), novobiocin (5 µg), rifampicin (5 µg), trimethoprim-sulfamethoxazole (25 µg) and vancomycin (30 µg) (MAST, Merseyside, UK). *S. aureus* ATCC33591 was used as a quality control.

#### DNA extraction from isolates

CoNS Chromosomal DNA was extracted by boiling method. Quality of extracted DNA was assessed by the Nanodrop ND–1000 (Nanodrop Technologies, Inc., Wilmington, DE, USA).

#### Detection of hemolysin genes

Identify of hemolysin encoding genes was performed by Multiplex PCR (*hla haem, hla/yidD epid* and *hlb_epid*) and single PCR (*hld_epi*) using specific primers (Additional file 1: Table S1), The PCR conditions included an initial denaturation at 94 °C for 4 min, followed by amplification; 30 cycles at 94 °C for 1 min, 58 °C for 1 min (45 °C-1 min for *hld_epi*), 72 °C for 1 min and 72 °C for 5 min. *hla_haem: S. haemolyticus* ATCC 29970, *hlb_epid: S. epidermidis* ATCC 12228 and *hla/yidD_epid: S. epidermidis* ATCC 12228, were used as control.

#### Detection of *mecA* gene

PCR assay was designed to amplify the *mecA* gene, using specific primers which are presented in Table 1. Amplification involved an initial denaturation at 95 °C for 10 min, followed by 35 cycles of denaturation (95 °C for 30 s), annealing (55 °C for 30 s), and extension (72 °C for 60 s), with a final extension step (72 °C for 7 min). *S. aureus* ATCC29247 was used as control.

**Table 1:** Prevalence of antibiotic-resistant strains among MR-CoNS and MS-CoNS

Antimicrobial agent	MR-CoNS (n = 50)			MS-CoNS (n = 41)		
	*S. epidermidis* (n = 26)	*S. haemolyticus* (n = 22)	*S. saprophyticus* (n = 2)	*S. epidermidis* (n = 23)	*S. haemolyticus* (n = 15)	*S. saprophyticus* (n = 3)
Cefoxitin	25	22	2	0	0	0
Trimethoprim-sulfamethoxazole	15	10	1	10	5	1
Erythromycin	4	8	0	9	4	1
Clindamycin	4	6	0	6	3	0
Chloramphenicol	8	7	0	6	2	0
Riphampin	0	0	1	1	1	0
Levofloxacin	1	3	0	4	1	2
Vancomycin	0	0	0	0	0	0
Gentamicin	10	6	0	2	2	0
Doxycycline	7	2	0	7	5	1
Novobiocin	0	0	2	0	0	3

*MR* methicillin-resistant, *CoNS* coagulase-negative *staphylococci*, *MS* methicillin-sensitive

#### Statistical analysis

Cramer’s V, Phi and Chi Square test were per-formed to assess of variables correlation. Phi and Cramer’s V have ranges from 0 to 1, where 1 indicates a significant association and 0 indicates no relationship. Interpretation of the Phi and Cramer’s V results; > 0; No or very weak, > 0.05 weak; > 0.10 moderate; > 0.15 strong; > 0.25 very strong. The Chi Square test was done by SPSS software version 20. P value < 0.05 was considered as statistically significant.

### Results

#### Isolation and prevalence of CoNS isolates

Of the 91 clinical isolates of CoNS, 49 (53.8), 37 (40.7%) and 5 (5.5%) isolates were recognized as *S. epidermidis, S. haemolyticus*, and *S. saprophyticus*, respectively. Isolates were recovered from blood 39 (42.9%), urine 33 (36.3%), catheter 13 (14.3%) and wound 6 (6.6%).

#### Antimicrobial susceptibility testing

The results of the antibiotic susceptibility testing of the 91 CoNS isolates, including 50 isolates (55%) of the Methicillin-resistant CoNS (MR-CoNS) and 41 isolates (45%) of the Methicillin-susceptible CoNS (MS-CoNS) are presented in Table 1. Multi-drug resistance was identified among 96% (48 isolates) of the MR-CoNS isolates and 63.4% (26 isolates) of the MS-CoNS isolates. Among MR-CoNS strains, the most frequent resistant, was to cefoxitin (98%). Among CoNS species, the highest resistance was observed for cefoxitin (53.8%,). None of the CoNS species was identified as being resistant to vancomycin.

#### Prevalence of hemolysin genes among CoNS isolates

The distribution of hemolysin genes among CoNS isolates is presented in Table 2. Our results showed a high frequency of hemolytic activity among CoNS strains isolated from blood, followed by urine, catheter, and wound, respectively. 80 isolates (87.9%) with *hla* gene, 57 isolates (62.6%) with *hla/yidD_epid*, 51 isolates (56%) with *hld* and 43 isolates (47.3%) with *hlb*. Only 15 isolates (16%) had one type of the hemolysin, included; 12, 1 and two *hla, hlb and hla/yidD* positive CoNS isolates, respectively. Two types of the hemolysins were identified in 32 (35%) of the strains, the greatest coexistence of genes was observed for the *hla *+ *hla/yidD* gene combination (14%), predominating followed by; *hla *+ *hld* = 12 (13%), *hla *+ *hlb *= 4 (0.04%), *hld *+* hla/yidD* = 2 (0.02%) and *hlb *+* hla/yidD* = 1 (0.01%). 14 (15%) of the strains were carrying three types of the hemolysins; *hla *+ *hlb *+ *hla/yidD*,*hlb *+ *hld *+ *hla/yidD* = 4 (0.04%) and *hla *+ *hld *+ *hla/yidD *= 3 (0.03%). All hemolysin genes were detected in 23 (25%) of the identified isolates.

**Table 2:** Prevalence of hemolysin encoding genes among various CoNS species and various clinical samples

Source					CoNS (n = 91)			
Catheter n (%)	Wound n (%)	Urine n (%)	Blood n (%)	Types of hemolysins	*S. epidermidis* n = 49))	*S. haemolyticus* n = 37))	*S. saprophyticus* (n = 5)	Total n (%)
10 (77)	4 (66.7)	29 (88)	37 (95)	*hla*	40 (81.6)	35 (94.6)	5 (100)	80 (87.9)
10 (77)	4 (66.7)	18 (54.5)	25 (64.1)	*hla/yiD*	34 (69.4)	23 (62.2)	0	57 (62.6)
6 (46.2)	4 (66.7)	15 (45.5)	18 (46.2)	*hlb*	26 (53.1)	17 (46)	0	43 (57.3)
7 (53.8)	4 (66.7)	18 (54.5)	22 (56.4)	*hld*	34 (69.4)	14 (37.8)	3 (60)	51 (56)

#### Prevalence of *mecA* gene among CoNS isolates

The distribution of *mec A* gene among CoNS isolates and the prevalence of hemolysin genes among MR-CoNS species are presented in Additional file 1: Table S2. *S. epidermidis* strains had the highest frequency of *mecA* gene. Among the MR-CoNS isolates, the *hla* gene was the most common with frequency of 90%, followed by the *hla_yiD* (60%), *hld* (58%) and *hlb* genes (48%).

#### Statistical analysis

Statistical analysis results are presented in Table 3. Statistically, a significant association between CoNS species and the occurrence of *hld* and *hla/yiD* genes was observed in this study. It was found a meaningful relationship between antibiotic resistance and the presence of the genes for alpha, delta and beta hemolysins.

**Table 3:** Statistical analysis results for determining possible relationship between the following variables and the presence of types of hemolysins genes

Types of hemolysins	*mecA* gene	Clinical sample	CoNS species	Cli	Ery	Levo	Novo	Rif
*hla*	P = 0.472	P = 0.122	P = 0.131	–	–	–	–	–
*hla_yiD*	P = 0.627	P = 0.545	*P = 0.009*	–	–	–	*P = 0.003* φ = 0.31	*P = 0.02* φ = 0.19
*hlb*	P = 0.962	P = 0.807	P = 0.075	*P = 0.009* φ = 0.28	–	–	*–*	
*hld*	P = 0.678	P = 0.954	*P = 0.014*	–	*P = 0.04* φ = 0.26	*P = 0.04* Φ = 0.21	–	

Statistically significant P values are in italic (P < 0.05)

*CoNS* coagulase-negative *staphylococci*, *Cli* clindamycin, *Ery* erythromycin, *Rif* rifampicin, *Levo* levofloxacin, *Novo* novobiocin

### Discussion

In the present study, *S. epidermidis* (53.8%) was the most clinically significant of the CoNS species and *S. haemolyticus* (40.7%) was identified as the second, which is in agreement with other reports [20–22]. The rate of the CoNS isolation was highest in blood (43%) followed by urine (36.3%), catheter (14.3%) and wound samples (6.6%). Aher et al, and Parashar et al, were also reported that the vast majority of CoNS species isolated from blood compared to urine [23, 24]. In our study, the highest resistance rate of the CoNS isolates was against methicillin (55%), followed by cefoxitin (53.8%). According to previous studies, which have found significant discrepancies in resistance ratio against cefoxitin with frequencies of 58% to 84.7% and high resistance to erythromycin (76.9%) and Trimethoprim-sulfamethoxazole (74.9%) [7, 25–27]. In current study, the resistance ratio to methicillin was determined in 50 (55%) of CoNS isolates; the findings were comparable to other studies [28–31], those reported resistance to methicillin with frequencies of 43.8%, 59.64%, 62.8%, and 73.3%, respectively. In this study, the prevalence rate of multiple antibiotic resistance was significantly higher among MR-CoNS isolates compared to MS-CoNS isolates which is similar to the results of Koksal et al, [31]. The results of our studies indicated that the *mecA* gene was identified in all CoNS isolates revealing phenotypical resistance to cefoxitin, and only one strain of *mecA*-positive *S. epidermidis* was phenotypically cefoxitin-susceptible, which is in agreement with the others [27, 32]. Like other studies [25, 33], this study show that all CoNS isolates were also completely sensitive to vancomycin. However, there are reports of the occurrence of decreased susceptibility to vancomycin in CoNS isolates [34, 35]. Out of the 91 CoNS isolates, the percentage of strains with hemolytic activity were the highest among *S. epidermidis (*53.8%), followed by *S. haemolyticus* (39.5%) which is in agreement with the findings of Cunha et al, and Pinheiro et al., [36, 37], but, in contrary with the findings of Akinkunmi et al., [20]. Among CoNS species, 94.6% and 81.6% of the *S. haemolyticus* and *S. epidermidis* strains were determined to be positive to the *hla* gene, respectively. The *hlb* gene was detected in 53% and 46% of the *S. epidermidis* and *S. haemolyticus* strains, respectively. On the other hand, according to Okee MS et al., the occurrence of *hlb gene* not observed in any of the CoNS species isolated and the *hla* gene found in only 20% of *S. epidermidis*, which is also in contrary to a similar survey carried out by Pinheiro et al, who demonstrated the emergence of the *hla* and *hlb* genes in 92.9% of the *S. epidermidis* strains and *hla* with the same frequency (91.7%) in *S. hemolyticus* strains [37, 38]. In this study, the largest number of the strains carrying the *hld* gene belonged to *S. epidermidis* with frequency of 69.4%, then *S. hemolyticus* (37.8%), according to Pinheiro et al, 95.3% of the *S. epidermidis* and none of the *S. haemolyticus* were carrying *hld* gene, [37]. In the comparative statistical analysis among the MR-CoNS and MS-CoNS isolates found no significant association between the emergence of the hemolysins and methicillin resistance (*p value* from 0.472 to 0.962). All the isolated MR-CoNS demonstrated hemolytic activity either alone or in combined forms, while only one of the MS-CoNS strains was nonhemolytic which belonged to a *S. haemolyticus* recovered from urine sample. Among MR-CoNS, the highest number of strains carrying hemolysin genes are isolated from the blood rather than urine. But in a similar study done by Motamedi et al, *S. aureus* hemolytic isolates were identified more in blood and ulcers compared to urine and catheter samples [39]. Regarding with the pattern of antimicrobial resistance in CoNS isolates found a significant association between the occurrence of *hld* gene and resistance to levofloxacin and erythromycin (P = 0.04, φ = 0.21 and P = 0.04, V = 0.26, respectively), the *hla/yidD* and resistance to rifampicin and novobiocin (P = 0.02, φ = 0.19 and P = 0.003, φ = 0.31, respectively), as well as between *hlb* and clindamycin resistance (P = 0.009, φ = 0.28). Our results showed that the frequency of hemolytic agent genes of *hla, hlb, hld* were higher in cefoxitin resistant isolates compared to susceptible ones. The analysis of our results demonstrated that there is no significant association between different clinical samples and hemolysins which is similar to the results of studies conducted by Corredor Arias et al., in *S. aureus* strains [40]. Arabestani et al., Lee et al., and Osman et al. have been proved significant associations between pathogenicity factors and antibiotic resistance [41–43].

### Conclusion

The results of this study demonstrated the significant role of antibiotic resistance in different infections due to increasing resistance to methicillin and the other antimicrobial agents among CoNS isolates. This study showed that the *hla* and *hla_yidD* genes and resistance to methicillin, cefoxitin, and trimethoprim-sulfamethoxazole antibiotics are widely distributed among the majority of CoNS isolated from human.

## Limitations

The number of bacteria isolates was rather low. If the number of bacteria was more, the results would be better and also we have to design another study which focus on gene expression of the resistance and virulence factors.

## Supplementary information

**Additional file 1: Table S1.** Primers used in this study. **Table S2.** The prevalence of *mecA* gene and types of hemolysins among MR-CoNS species.

## Data Availability

The Additional files included Additional file 1: Tables S1 and S2.

## Abbreviations

CoNS: Coagulase-negative *staphylococci*
PFT: Pore-forming cytotoxin
BHI: Brain Heart Infusion
DAD: Disk agar diffusion
CLSI: Clinical and Laboratory Standards Institute
MS-CoNS: Methicillin-susceptible CoNS

## References

[1] Soumya K, Philip S, Sugathan S, Mathew J, Radhakrishnan E (2017). Virulence factors associated with coagulase negative *Staphylococci* isolated from human infections. 3 Biotech.

[2] Azih A, Enabulele I (2013). Species distribution and virulence factors of coagulase negative *Staphylococci* isolated from clinical samples from the University of Benin Teaching hospital, Edo State, Nigeria. J Nat Sci Res.

[3] Nanoukon C, Argemi X, Sogbo F, Orekan J, Keller D, Affolabi D (2017). Pathogenic features of clinically significant coagulase-negative *staphylococci* in hospital and community infections in Benin. Int J Med Microbiol.

[4] Mehri H, Jahanbakhsh R, Shakeri F, Ardebili A, Behnampour N, Khodabakhshi B (2017). Investigation of glycopeptide susceptibility of coagulase-negative *staphylococci* (CoNS) from a tertiary care hospital in Gorgan, northern Iran. Arch Pediatr Infect Dis..

[5] Xu Z, Mkrtchyan HV, Cutler RR (2015). Antibiotic resistance and mecA characterization of coagulase-negative *staphylococci* isolated from three hotels in London, UK. Front Microbiol.

[6] May L, Klein EY, Rothman RE, Laxminarayan R (2014). Trends in antibiotic resistance in coagulase-negative *staphylococci* in the United States, 1999 to 2012. Antimicrob Agents Chemother.

[7] Shrestha LB, Bhattarai NR, Khanal B (2017). Antibiotic resistance and biofilm formation among coagulase-negative *staphylococci* isolated from clinical samples at a tertiary care hospital of eastern Nepal. Antimicrob Resist Infect Control..

[8] Chovanová R, Mikulášová M, Vaverková Š (2013). In vitro antibacterial and antibiotic resistance modifying effect of bioactive plant extracts on methicillin-resistant *Staphylococcus epidermidis*. Int J Microbiol.

[9] Moraveji Z, Tabatabaei M, Aski HS, Khoshbakht R (2014). Characterization of hemolysins of *Staphylococcus* strains isolated from human and bovine, southern Iran. Iran J Vet Res.

[10] Gemmell C, Roberts E (1974). Toxins and enzymes of coagulase-negative *staphylococci* isolated from human infections. J Hyg Epidemiol Microbiol Immunol.

[11] Qiu J, Niu X, Dong J, Wang D, Wang J, Li H (2012). Baicalin protects mice from *Staphylococcus aureus* pneumonia via inhibition of the cytolytic activity of α-hemolysin. J Infect Dis.

[12] Berube B, Wardenburg J (2013). *Staphylococcus aureus* α-toxin: nearly a century of intrigue. Toxins..

[13] Ebrahimi A, Akhavan Taheri M (2009). Characteristics of *staphylococci* isolated from clinical and subclinical mastitis cows in Shahrekord, Iran. Iran J Vet Res.

[14] Oliveira D, Borges A, Simões M (2018). *Staphylococcus aureus* toxins and their molecular activity in infectious diseases. Toxins..

[15] Burnside K, Lembo A, de los Reyes M, Iliuk A, BinhTran N-T, Connelly JE (2010). Regulation of hemolysin expression and virulence of *Staphylococcus aureus* by a serine/threonine kinase and phosphatase. PLoS ONE..

[16] Zhang YQ, Ren SX, Li HL, Wang YX, Fu G, Yang J (2003). Genome-based analysis of virulence genes in a non-biofilm-forming Staphylococcus epidermidis strain (ATCC 12228). Mol Microbiol.

[17] Marconi C, Cunha M, Araújo J, Rugolo L (2005). Standardization of the PCR technique for the detection of delta toxin in *Staphylococcus* spp. J Venom Anim Toxins Incl Trop Dis.

[18] Cunha CR (2008). Toxigenicity in *Staphylococcus aureus* and coagulase-negative *staphylococci*: epidemiological and molecular aspects. Microbiol Insights..

[19] Wayne PA. CLSI In; Performance standards for antimicrobial susceptibility testing. 29th ed. CLSI supplement M100, Clinical and Laboratory Standards Institute; 2019.

[20] Akinkunmi E, Lamikanra A (2010). Species distribution and antibiotic resistance in coagulase-negative *staphylococci* colonizing the gastrointestinal tract of children in Ile-Ife, Nigeria. Trop J Pharm Res.

[21] Begum ES, Anbumani N, Kalyani J, Mallika M (2011). Prevalence and antimicrobial susceptibility pattern of coagulase-negative *Staphylococcus*. Int J Med Public Health..

[22] Al Tayyar IA, Al-Zoubi MS, Hussein E, Khudairat S, Sarosiekf K (2015). Prevalence and antimicrobial susceptibility pattern of coagulase-negative *staphylococci* (CoNS) isolated from clinical specimens in Northern of Jordan. Iran J Microbiol.

[23] Aher CS (2014). The isolation pattern, species distribution and antibiotic susceptibility profile of coagulase negative *Staphylococci*: emerging opportunistic pathogens. Int J Biomed Adv Res.

[24] Parashar S. Significance of coagulase negative *Staphylococci* with special reference to species differentiation and antibiogram. Indian Med Gaz. 2014:255–8.

[25] Goudarzi M, Seyedjavadi SS, Goudarzi H, Boromandi S, Ghazi M, Azad M (2014). Characterization of coagulase-negative *staphylococci* isolated from hospitalized patients in Tehran, Iran. J Paramed Sci.

[26] Fowoyo P, Ogunbanwo S (2017). Antimicrobial resistance in coagulase-negative *staphylococci* from Nigerian traditional fermented foods. Ann Clin Microbiol Antimicrob.

[27] Chajęcka-Wierzchowska W, Zadernowska A, Nalepa B, Sierpińska M, Łaniewska-Trokenheim Ł (2015). Coagulase-negative *staphylococci* (CoNS) isolated from ready-to-eat food of animal origin–phenotypic and genotypic antibiotic resistance. Food Microbiol.

[28] Perveen I, Majid A, Knawal S, Naz I, Sehar S, Ahmed S (2013). Prevalence and antimicrobial susceptibility pattern of methicillin-resistant *Staphylococcus aureus* and coagulase-negative *Staphylococci* in Rawalpindi, Pakistan. J Adv Med Med Res.

[29] Gilani M, Usman J, Latif M, Munir T, Gill MM, Anjum R (2016). Methicillin resistant coagulase negative *staphylococcus*: from colonizer to a pathogen. Pak J Pharm Sci..

[30] Mehdinejad M, Sheikh AF, Jolodar A (2008). Study of methicillin resistance in *Staphylococcus aureus* and species of coagulase negative *staphylococci* isolated from various clinical specimens. Pak J Med Sci..

[31] Koksal F, Yasar H, Samasti M (2009). Antibiotic resistance patterns of coagulase-negative *staphylococcus* strains isolated from blood cultures of septicemic patients in Turkey. Microbiol Res.

[32] Vyletelova M, VlkoVá H, Manga I (2011). Occurrence and characteristics of methicillin resistant *Staphylococcus aureus* and methicillin resistant coagulase-negative *staphylococci* in raw milk manufacturing. Czech J Food Sci..

[33] Keim LS, Torres-Filho SR, Silva PV, Teixeira LA (2011). Prevalence, aetiology and antibiotic resistance profiles of coagulase negative *staphylococci* isolated in a teaching hospital. Braz J Microbiol.

[34] Pinheiro L, Brito CI, Pereira VC, Oliveira Ad, Camargo CH, Cunha MdLRd (2014). Reduced susceptibility to vancomycin and biofilm formation in methicillin-resistant *Staphylococcus epidermidis* isolated from blood cultures. Memorias do Instituto Oswaldo Cruz..

[35] Mashaly GES, El-Mahdy RH (2017). Vancomycin heteroresistance in coagulase negative *Staphylococcus* blood stream infections from patients of intensive care units in Mansoura University Hospitals, Egypt. Ann Clin Microbiol Antimicrob..

[36] Cunha MdLRd, Rugolo LMSdS, Lopes CAdM (2006). Study of virulence factors in coagulase-negative *staphylococci* isolated from newborns. Memórias do Instituto Oswaldo Cruz..

[37] Pinheiro L, Brito C, de Oliveira A, Martins P, Pereira V, da Cunha M (2015). *Staphylococcus epidermidis* and *Staphylococcus haemolyticus*: molecular detection of cytotoxin and enterotoxin genes. Toxins..

[38] Okee MS, Joloba ML, Okello M, Najjuka FC, Katabazi FA, Bwanga F (2012). Prevalence of virulence determinants in *Staphylococcus epidermidis* from ICU patients in Kampala, Uganda. J Infect Dev Ctries.

[39] Motamedi H, Asghari B, Tahmasebi H, Arabestani MR (2018). Identification of hemolysine genes and their association with antimicrobial resistance pattern among clinical isolates of *Staphylococcus aureus* in West of Iran. Adv Biomed Res.

[40] Corredor Arias LF, Espinal L, Samara J, Moncayo Ortiz JI, Santacruz Ibarra JJ, Álvarez Aldana A (2016). Relationship between super antigenicity, antimicrobial resistance and origin of *Staphylococcus aureus* isolated. Colombia Médica..

[41] Arabestani MR, Rastiyani S, Alikhani MY, Mousavi SF (2018). The relationship between prevalence of antibiotics resistance and virulence factors genes of MRSA and MSSA strains isolated from clinical samples, West Iran. Oman Med J.

[42] Lee GY, Lee H-H, Hwang SY, Hong J, Lyoo K-S, Yang S-J (2019). Carriage of *Staphylococcus schleiferi* from canine otitis externa: antimicrobial resistance profiles and virulence factors associated with skin infection. J Vet Sci.

[43] Osman K, Alvarez-Ordóñez A, Ruiz L, Badr J, ElHofy F, Al-Maary KS (2017). Antimicrobial resistance and virulence characterization of *Staphylococcus aureus* and coagulase-negative *staphylococci* from imported beef meat. Ann Clin Microbiol Antimicrob.

