# Supplementary material for: Identification of hemolysin encoding genes and their association with antimicrobial resistance pattern among clinical isolates of coagulase-negative Staphylococci
Source: BMC Res Notes. 2020 Feb 10;13:68. doi: 10.1186/s13104-020-4938-0 (PMC7011549; doi:10.1186/s13104-020-4938-0)
Supplement: Supplementary file 1 — Additional file 1: Table S1. Primers used in this study. Table S2. The prevalence of mecA gene and types of hemolysins among MR-CoNS species. [file 13104_2020_4938_MOESM1_ESM.docx]

**Table S1.**

| **References** | **amplicon / product**  **size (bp)** | **Primer sequences (5' to 3')** | **Gene targets** |
| --- | --- | --- | --- |
| [1] | 72 | F: TGGGCCATAAACTTCAATCGC  R: ACGCCACCTACATGCAGATTT | *hla_haem* |
| [1] | 160 | F: TTTCKCCACTTACACCMCC  R: GGAACAGGATCAAAGCCACCT | *hla_yiD_epi* |
| [1] | 541 | F: TGGTGGCGTTGGTATTGTGA  R: ACCCCAAGATTTCACGGACC | *hlb_epi* |
| [1] | 444 | F: ATGGCAGCAGATATCATTTC  R: CGTGAGCTTGGGAGAGAC | *hld_epi* |
| **[2]** | 162 | F: TCCAGATTACAACTTCACCAGG  R: CCACTTCATATCTTGTAACG | *mec A* |

**Table S2.**

|  | *mecA* gene(n=50) | | |  |  |
| --- | --- | --- | --- | --- | --- |
| *Total*  *n(%)* | *S. saprophyticus*(n=2) | *S. haemolyticus*(n=22) | *S. epidermidis*(n=26) |  | Types of hemolysins |
| 45(90) | 2 | 21 | 24 |  | *hla* |
| 30(60) | 0 | 14 | 16 |  | *hla_yid* |
| 24(48) | 0 | 8 | 14 |  | *hlb* |
| 29(58) | 2 | 6 | 20 |  | *hld* |

**References**

1. Chessa D, Ganau G, Spiga L, Bulla A, Mazzarello V, Campus GV, et al. *Staphylococcus* *aureus* and *Staphylococcus* *epidermidis* virulence strains as causative agents of persistent infections in breast implants. PLoS One. 2016;**11**(1):e0146668.

2. Murugesan S, Perumal N, Mahalingam SP, Dilliappan SK, Krishnan P. Analysis of antibiotic resistance genes and its associated SCCmec types among nasal carriage of methicillin resistant coagulase negative *Staphylococci* from community settings, Chennai, Southern India. Journal of clinical and diagnostic research: JCDR. 2015;**9**(8):DC01.
